# Self-Assembly of Free-Standing LiMn_2_O_4_-Graphene Flexible Film for High-Performance Rechargeable Hybrid Aqueous Battery

**DOI:** 10.3390/ma11071056

**Published:** 2018-06-21

**Authors:** Guanghui Yuan, Ting Huang, Ying Kou, Zhen Ji, Yan Zhao

**Affiliations:** 1 Department of Chemistry and Chemical Engineering, Research Centre of New Materials, Ankang University, Ankang 725000, China; tingyu2008jh@126.com (T.H.); streamlet2000@163.com (Y.K.); jizhen@aku.edu.cn (Z.J.); 2 Synergy Innovation Institute of GDUT, Heyuan 517000, China

**Keywords:** LiMn_2_O_4_-graphene, flexible film, rechargeable hybrid aqueous batteries, electrochemical performance

## Abstract

A novel LiMn_2_O_4_-graphene flexible film is successfully prepared by facile vacuum filtration technique. LiMn_2_O_4_ nanowires with diameters of 50–100 nm are distributed homogeneously on graphene sheet matrix. Used as cathode in rechargeable hybrid aqueous batteries, the LiMn_2_O_4_-graphene film exhibits enhanced electrochemical performance in comparison to LiMn_2_O_4_-graphene powder. The LiMn_2_O_4_-graphene film shows stable 13.0 mAh g^−1^ discharge capacity after 200 cycles at 1.0 C, benefitting from the presence of graphene with strong conductivity and large pore area in this free-standing film. This synthetic strategy for a free-standing film can provide a new avenue for other flexible materials and binder-free electrodes.

## 1. Introduction

The new generation of the electronic equipment, such as light wearable electronic devices and electric vehicles with high energy density batteries, is accelerating the development of rechargeable batteries [1,2]. The traditional rechargeable lithium-ion batteries with organic electrolyte are facing fiercer and fiercer challenge due to their high cost and low safety [3,4]. Recently, aqueous rechargeable lithium batteries have attracted increasing attention in large-scale energy storage systems due to their lower toxicity, lower cost and better safety, thanks to water solutions instead of organic electrolytes [5]. Among these, the rechargeable hybrid aqueous battery (ReHAB) has been attracting increasing attention [6].

The ReHAB is composed of a zinc metal anode and a traditional cathode (such as LiFePO_4_ and LiMn_2_O_4_), in which the Zn anode undergoes the reversible redox reaction, while the LiFePO_4_ or LiMn_2_O_4_ cathode, for instance, undergoes lithium intercalation/de-intercalation. The electrochemical reactions can be written as follows.

Anode: *x*Zn^2+^*+ 2x*e^−^ ⇄ *x*Zn

Cathode: *2*LiMn_2_O_4_ ⇆ *2*Li_1−x_Mn_2_O_4_ + *2x*Li^+^ + *2x*e^−^ (0 ≤ *x* ≤ 1)

Recent reports have presented Zn/LiCl + ZnCl_2_/LiMn_2_O_4_, Zn/LiCH_3_COO + Zn(CH_3_COO)_2_/LiFePO_4_, and Zn/LiCH_3_COO + Zn(CH_3_COO)_2_/MnO_2_ ReHABs with different cathodes and electrolytes [7,8,9]. Limited by poor electronic conductivity, low lithium ion diffusion rate and drastic volume change, the electrochemical properties of pristine LiFePO_4_ or LiMn_2_O_4_ cathodes often deteriorate drastically with increasing of charge-discharge rates. To overcome these issues, one of the effective strategies is anchoring the active LiFePO_4_ or LiMn_2_O_4_ particles into various porous carbon matrixes to enhance the electrical conductivity and suppress the volume change during cycling [10,11,12].

Among various carbon-based materials, graphene or reduced graphene oxide is always intensively investigated, due to the large specific surface area, extraordinary electronic transport property and high electrochemical stability. Some work has discussed the positive effect on the electrochemical properties of graphene-based LiFePO_4_ or LiMn_2_O_4_ composites [12,13,14]. It is worth noting that most of this work is focused on powder graphene composites and their application in lithium rechargeable batteries with organic electrolyte. Recently, research into flexible films as binder-free electrodes for rechargeable batteries has developed rapidly to power new applications, such as light and soft wearable electronic devices [15,16]. Compared to carbon nanotubes and other carbon-based materials, it is most convenient to make graphene into flexible film because of its layered structure [17]. However, few works have discussed graphene-based flexible film electrodes for rechargeable hybrid aqueous batteries.

Herein, a free-standing LiMn_2_O_4_-graphene flexible film is designed and prepared by a facile vacuum filtration method, and its electrochemical performance is investigated for the first time in ReHAB. Compared to the LiMn_2_O_4_-graphene powder prepared by simple physical mixing and the slurry casting technique, the LiMn_2_O_4_-graphene film exhibits an amazingly stable cycling ability and enhanced rate performance.

## 2. Materials and Methods

### 2.1. Materials Preparation

Graphene oxide (GO) was synthesized by natural flake graphite according to previous work [18]. LiMn_2_O_4_ powder was prepared by the following process. 0.095 g KMnO_4_ was dissolved in 25 mL deionized (DI) water and underwent ultrasonic radiation for 0.5 h to form the first solution. The second solution was arranged by dissolving 0.220 Mn(CH_3_COO)_2_∙4H_2_O in 25 mL DI water. The mixture of the two solutions was transferred and sealed in a 60 mL Teflon autoclave for 20 h at 160 °C. After the hydrothermal reaction, black MnO_2_ powder was collected, followed by centrifugation, washing with ethanol and DI water, and drying in vacuum. The prepared MnO_2_ and LiOH·H_2_O powders with a molar ratio of 2:1 were ground with ethanol for 1 h. After air solid-state reaction at 400 °C for 8 h and 750 °C for 10 h, the expected LiMn_2_O_4_ powder (LMO) was prepared.

**Figure 1:**
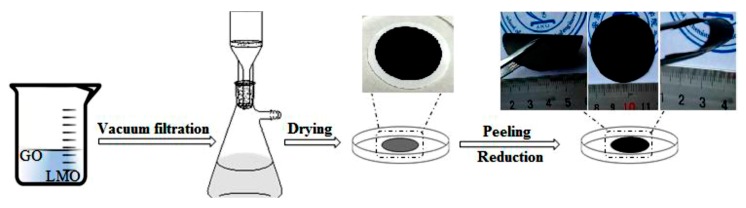
Schematic for the preparation process of the LiMn_2_O_4_-graphene film.

The flexible LiMn_2_O_4_-graphene film was manufactured by an ordinary vacuum filtration method followed by a thermal reduction process. Typically, 30 mg GO was dispersed in 10 mL deionized water and underwent ultrasonic operation for 2 h to a uniform brown GO suspension. 20 mg LiMn_2_O_4_ powder was secondly dispersed into the GO suspension to undergo ultrasonic treatment for 2 h. The suspension was then filtered through a filter membrane under vacuum. The flexible film was peeled off carefully from the membrane after washing, drying and immersing in acetone for 15 min. After being heat treated in air at 220 °C for 2 h to reduce GO into graphene (GN), the final flexible LiMn_2_O_4_-graphene film was obtained, labeled as LMO/GN-F. The preparation procedure and photos of the flexible film are shown in Figure 1. For comparison, the LiMn_2_O_4_-graphene powder (LMO/GN-P) was prepared by simple physical ball-milling.

### 2.2. Material Characterization

The crystalline phases of the as-prepared samples were determined by X-ray powder diffraction (XRD, D8 ADVANCE, Bruker, Billerica, MA, USA) equipped with Cu Kα radiation (λ = 0.15418 nm) at a scanning rate of 0.02° s^−1^ in 10~70°. The content of LiMn_2_O_4_ in the LiMn_2_O_4_-graphene powder and the LiMn_2_O_4_-graphene film were confirmed by thermoanalyzer (DSC–TGA; SDT Q600, TA Company, Boston, MA, USA) with air flow from room temperature to 600 °C at 10 °C min^−1^. To determine the pore volumes and specific surface areas of the prepared films, Brunauere Emmette Teller (BET) and Barrette Joynere Halenda (BJH) methods were carried out using nitrogen adsorption. The surface morphologies of the samples were examined by field emission scanning electron microscopy (SEM, Quanta FEG-400) and transmission electron microscopy (TEM, FEI-Tecnai G^2^-F20 S-TWIN) techniques.

### 2.3. Electrochemical Measurements

The free-standing LMO/GN-F electrodes were prepared by cutting the flexible LMO/GN film into 10 mm circles directly. The compared LMO/GN-P electrodes were prepared by brushing the n-methyl-2-pyrrolidinone slurry containing LMO/GN powder, acetylene black, and polyvinylidene fluoride (80:10:10 wt %) on 10 mm stainless steel foil, followed by vacuum drying at 110 °C for one night. The stainless steel foil was pressed at 10 MPa to achieve superior contact between the active material and the current collector. The CR2025 coin cells were assembled using Zn metal as anode, 0.5 M LiCH_3_COO and 0.5 M Zn(CH_3_COO)_2_ aqueous solution as electrolyte, absorbent glass mat wet as separator, and the LMO/GN-F or LMO/GN-P electrode as cathode.

The cyclic voltammetry (CV) was carried out on a CHI 660D electrochemical workstation at a scan rate of 0.15 mV s^−1^ in the potential range of 1.35–2.15 V vs. Zn/Zn^2+^. The galvanostatic charge-discharge tests were arranged on a LAND battery program-controlled tester in a cut-off potential window of 1.45–2.05 V. Electrochemical impedance spectroscopy (EIS) was performed by using the CHI 660D electrochemical workstation with a frequency range from 0.01 to 100 kHz.

## 3. Results and Discussion

**Figure 2:**
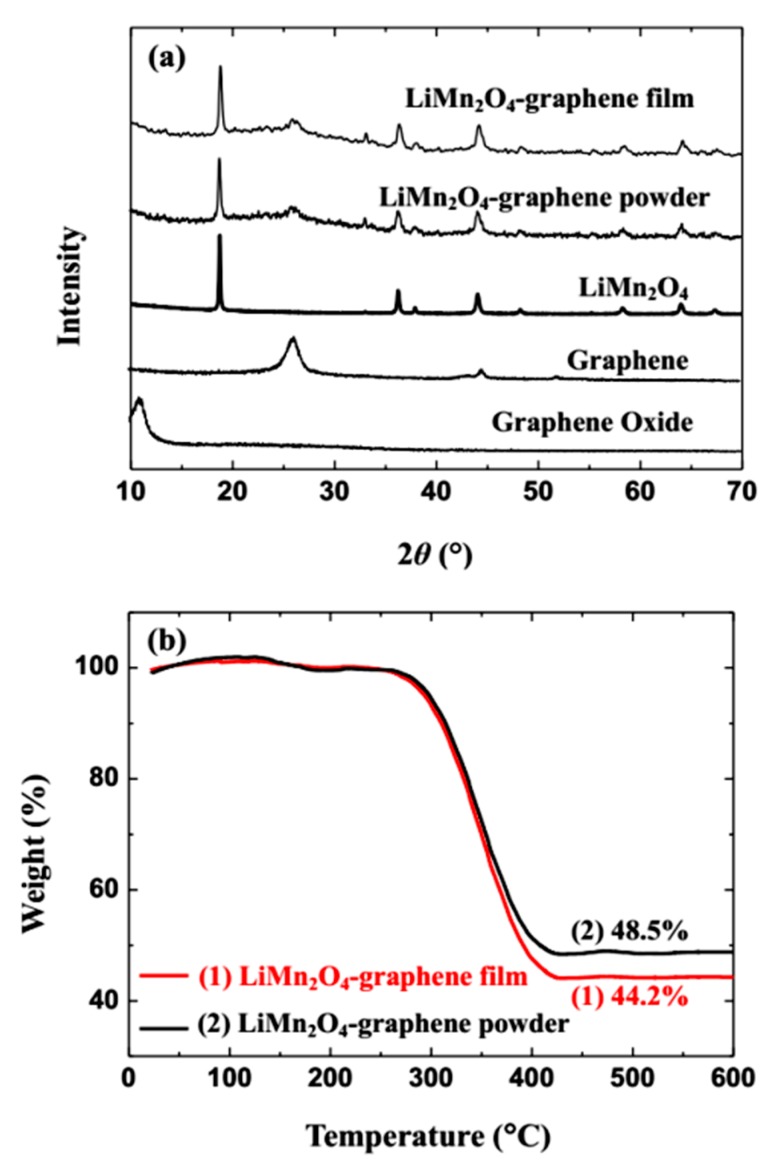
XRD patterns of samples (**a**), and thermogravimetric curves of LiMn_2_O_4_-graphene film and LiMn_2_O_4_-graphene powder (**b**).

Figure 2a shows the X-ray diffraction (XRD) patterns of the as-prepared samples. The XRD patterns of GO displays only one obvious peak centered at around 11.5°, which can be attributed to the (002) reflection of graphene oxide. The XRD patterns of GN display one obvious peak centered at around 25.6° and one weak peak at around 43.6°, which can be attributed to the (002) and (100) reflections of graphene, respectively [19]. The XRD pattern of LMO used in our experiment shows the typical reflection pattern of cubic spinel LiMn_2_O_4_ with a space group of Fd3m [20]. The XRD pattern of LMO/GN-F exhibits the characteristic features of spinel LiMn_2_O_4_ and a broad typical peak of graphene at around 25.6°, indicating that there are no phase transformations for LMO in the LMO/GN film. No detectable peak at around 11.5° is observed, indicating that graphene oxide is reduced completely to graphene during the experiment process. The XRD pattern of LMO/GN-P exhibits a very similar shape with the XRD pattern of LMO/GN-F, indicating that the LiMn_2_O_4_-graphene composite can be made successfully by the physical ball-milling technique. The LMO contents in the LMO/GN film and as-prepared LMO/GN powder are estimated by TGA under air atmosphere with a heating rate of 10 °C min^−1^. Both LMO/GN-F and LMO/GN-P TGA curves in Figure 2b show only one drastic weight loss from around 300 °C to 450 °C. From 450 °C upon 600 °C, the two TGA curves remain approximately unchanged. The results indicate that the carbon component in LMO/GN-F and LMO/GN-P is completely burned in air flow [21,22]. The two TGA curves remain unchanged from 450 °C to 600 °C, showing that the LMO in the two samples has no phase transformations during the heat treatment. The LMO/GN-F and LMO/GN-P exhibit around 44.2% and 48.5% LMO content, respectively. The similar amounts of LMO content in the different samples can eliminate the influence of different content on electrochemical performance.

**Figure 3:**
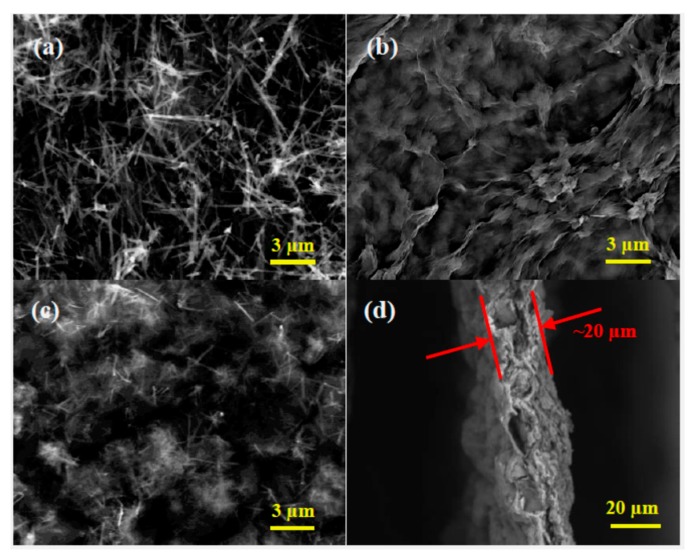
SEM images of LiMn_2_O_4_ (**a**), graphene film (**b**), surface (**c**), and fracture edge (**d**) of LiMn_2_O_4_-graphene film.

**Figure 4:**
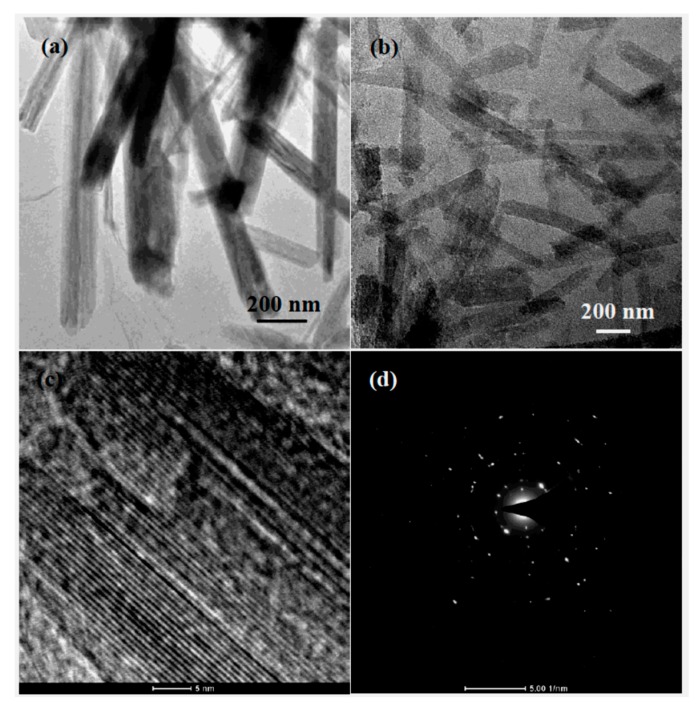
TEM images of LiMn_2_O_4_ (**a**), gradually enlarged TEM images (**b**,**c**), and SAED pattern (**d**) of the LiMn_2_O_4_-graphene film.

The morphology of the synthesized LMO, GN film and LMO/GN film are measured by SEM (Figure 3). The LMO nanowires are 5~10 μm in length (Figure 3a). There is a slight agglomeration among LMO nanowires. The pristine GN film has curved and wrinkled surface morphology (Figure 3b). The LMO/GN film has a similar wrinkled surface to GN film, except that LMO nanowires are homogeneously distributed in the surface (Figure 3c). The thickness of the LMO/GN film is about 20 μm (Figure 3d). The fracture edge of this film displays layer-by-layer stacking of graphene sheets. To further determine the size of LMO nanowires and their distribution in the LMO/GN film, the samples are characterized by TEM. The pristine LMO nanowires have 50–100 nm in diameter (Figure 4a). The LMO nanowires are anchored uniformly in the LMO/GN film (Figure 4b) and exhibit the clear lattice fringes of LMO surrounded by unclear graphene polycrystalline lattice (Figure 4c). The selected area electron diffraction (SAED) pattern of the LMO (Figure 4d) is composed of spotted rings indexed to the (111), (311), (400), (222) and (331) planes of cubic LiMn_2_O_4_ inside and out. The SEM, TEM images and the XRD patterns in Figure 2a clearly reveal that the LMO/GN film is prepared successfully by our facile vacuum filtration method.

**Figure 5:**
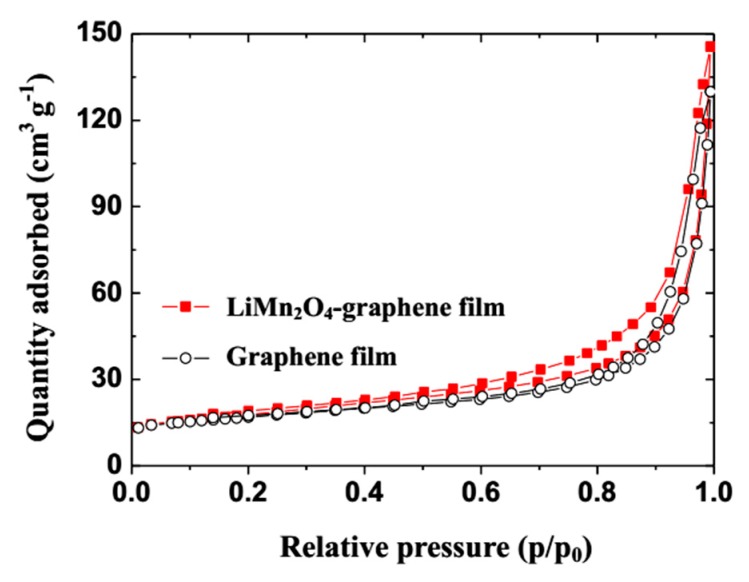
The N_2_ isotherm adsorption and desorption curves of the graphene film and the LiMn_2_O_4_-graphene film.

In order to determine the specific surface area and pore volumes of the GN film and LMO/GN film, the N_2_ adsorption and desorption isotherms are measured and shown in Figure 5. The BET specific surface area of the LMO/GN film is calculated to be 60.4 cm^2^ g^-1^, which is obviously higher than that of the pristine GN film (39.6 cm^2^ g^-1^). This result can be attributed to the presence of LMO nanowires on or in the surface of the GN support. The LMO nanowires intercalating into the GN nanosheets may not only result in more pores, but also prevent the aggregation of the GN nanosheets. In the meantime, the adsorption of the GN nanosheets can prevent the aggregation of the LMO nanowires. Therefore, the total pore volume of the LMO/GN film is 0.30 cm^3^ g^-1^, which is higher than that of the pristine GN film (0.21 cm^3^ g^-1^). Combining the result with TEM measurements, the wrinkled surface morphology, large pore volume and specific surface area of the LMO/GN film can permit easy access for electrons and ions to the electrode/electrolyte and accommodate the volume change of the LMO nanowires during the charge and discharge process [23].

**Figure 6:**
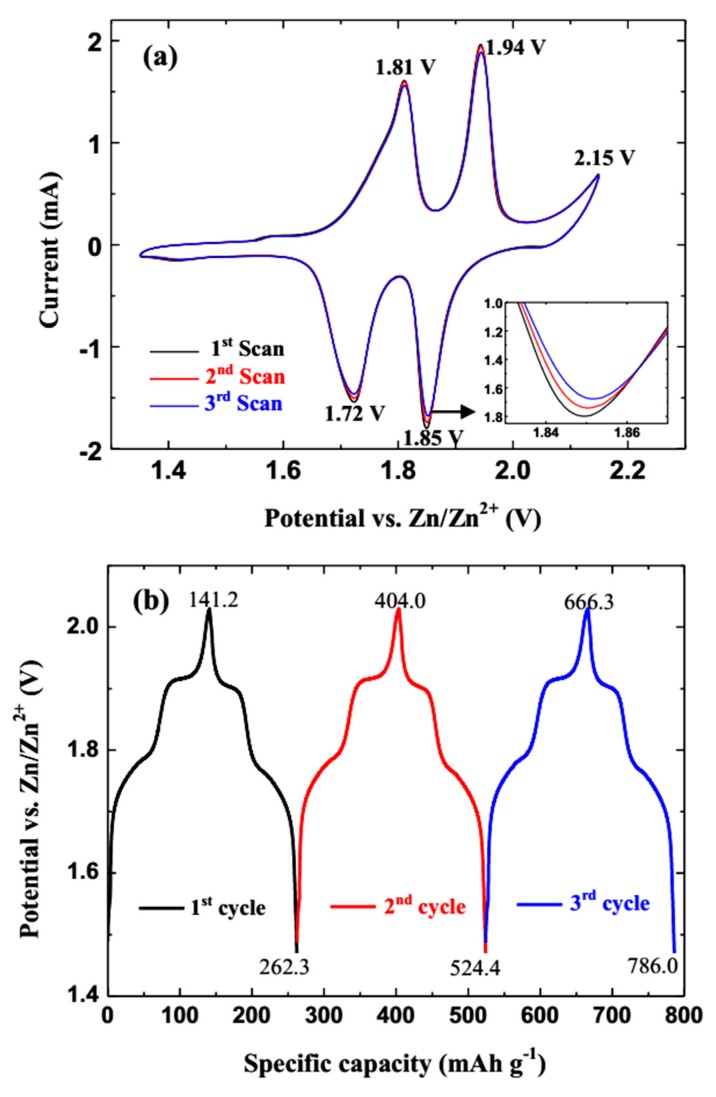
CV curves (**a**) and the initial charge-discharge profiles (**b**) of the LiMn_2_O_4_-graphene film cathode at 0.2 C. Inset in (**a**) is the enlarged detail.

The galvanostatic discharge-charge and CV tests are important challenging and key aspects for ReHAB applications. Aiming to examine the electrochemical performance of the LMO/GN-F electrode sufficiently, the LMO/GN-F as cathode material for ReHAB is examined by CV and galvanostatic discharge-charge tests, as shown in Figure 6. All the CV curves over three cycles exhibit two obvious pairs of oxidation and reduction peaks between 1.6 and 2.0 V vs. Zn/Zn^2+^, corresponding to the two-step lithium de/intercalation of the LMO/GN-F electrode (Figure 6a). In detail, the oxidation peak at 1.81 V is corresponding to the deintercalation of lithium ions from the spinel LiMn_2_O_4_ structure until half of the 8a sites are empty in Li*_x_*Mn_2_O_4_ (0.5 ≤ *x* ≤ 1). The subsequent oxidation peak at 1.94 V corresponds to the continued deintercalation of lithium ions until all of the 8a sites are empty. At this point, LiMn_2_O_4_ is fully oxidized to λ-MnO_2_. The anodic peak at 2.15 V vs. Zn/Zn^2+^ is assigned to O_2_ evolution due to the water decomposition [6]. The reduction peak at 1.85 V is assigned to the intercalation of lithium ions into each available tetrahedral site (8a) in λ-MnO_2_, until half of the sites are filled in Li*_x_*Mn_2_O_4_ (0 < *x* < 0.5). The other reduction peak at 1.72 V is associated with the lithium ions filling the remaining empty 8a sites to form Li*_x_*Mn_2_O_4_ (0.5 ≤ *x* ≤ 1) [24,25]. As clearly shown in the inset in Figure 6a, the peak intensity weakens slightly with increase in the CV test cycle, which could be due to the small attenuation of electrochemical activity. Figure 6b illustrates the first three charge-discharge profiles of the LMO/GN-F electrode at 0.5 C. According to the CV data, the potential of the charge-discharge process is restricted from 1.45 V to 2.05 V vs. Zn/Zn^2+^ to avoid the water decomposition. As shown in Figure 6b, all three curves have two well-defined plateaus at about 1.76 V and 1.92 V vs. Zn/Zn^2+^ in the charge and discharge profiles corresponding to the two-step lithium de/intercalation mechanism of LMO/GN-F electrode, which is confirmed by the CV data. The specific discharge capacities of LMO/GN-F electrode in the first three cycles are 121.1 (262.3–141.2), 120.4 (524.4–404.0) and 119.7 (786.0–666.3) mAh g^−1^, respectively. The initial coulombic efficiency of LMO/GN-F electrode is around 85.7%. The low coulombic efficiency may be caused by the activation of the fresh electrode and the irreversible side reactions during the initial charge and discharge process.

**Figure 7:**
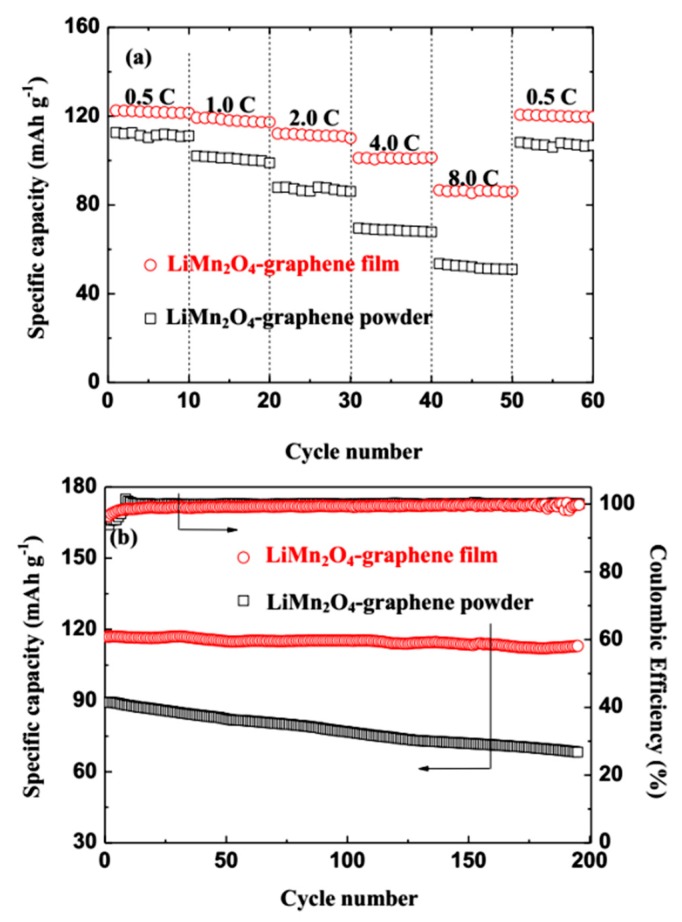
Rate capability (**a**) and cycle performance at 1C (**b**) of the LiMn_2_O_4_-graphene powder and LiMn_2_O_4_-graphene film cathodes.

To further examine the effect of GN in improving the electrochemical performance sufficiently, the electrodes containing LMO/GN-P or LMO/GN-F as cathodes for ReHABs are tested for C-rate and cycle ability for comparison (Figure 7). As shown in Figure 7a, as the C-rate increases stepwise, the specific discharge capacities of both LMO/GN-P and LMO/GN-F electrodes decrease obviously, which is due to the diffusion-controlled kinetics of the lithium de/intercalation reactions [26]. Compared to LMO/GN-P, the rate performance of the LMO/GN-F is significantly improved (Figure 7a). In detail, for LMO/GN-F, the reversible discharge capacities of 122.5, 119.3, 112.1, 101.1 and 86.6 mAh g^−1^ are acquired at 0.5, 1.0, 2.0, 4.0 and 8.0 C, respectively. The returning of the C-rate back to 0.5 C can recover the discharge capacity of 120.6 mAh g^−1^, indicating robustness of the LMO/GN flexible film. The long-term cyclabilities of the two electrodes are shown in Figure 7b. Both discharge capacities and capacity retentions of the LMO/GN-F electrode are obviously better than those of the LMO/GN-P electrode. After 200 cycles at 1.0 C, the discharge capacities of LMO/GN-F and LMO/GN-P electrodes are 113.0 mAh g^−1^ and 68.3 mAh g^−1^. The corresponding capacity retentions are 94.8% and 76.7%, respectively. The coulombic efficiencies of both electrodes reached 99% after a few activated cycles. All the data indicate that the LiMn_2_O_4_-graphene film exhibits an amazingly stable cycling ability and enhanced rate performance.

**Figure 8:**
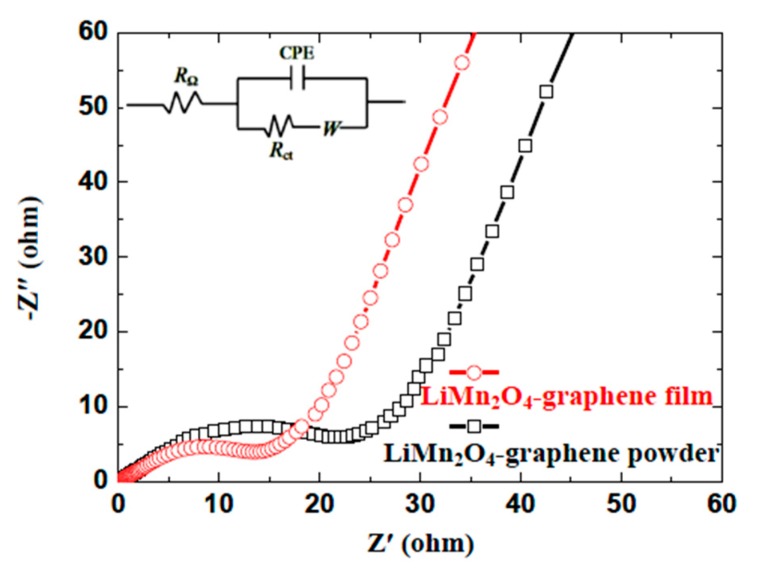
Nyquist plots of the LiMn_2_O_4_-graphene film and LiMn_2_O_4_-graphene powder cathodes after the first cycle showing the whole frequency region of 100 kHz to 0.01 Hz.

Benefitting from the vacuum filtration method, the LMO nanowires and graphene sheets in LMO/GN-F contact each other more closely than in LMO/GN-P prepared by simple physical mixing. The improvement of electrochemical performance is mainly attributed to the positive effects of graphene in enhancing electronic conductivity [27], decreasing the LMO nanowires agglomeration and handling the volume expansion of LMO during charge-discharge cycles. The positive influence of the LMO/GN-F electrode on charge transfer behavior and conductivity of the system can be proved by the EIS measurements (Figure 8). As shown in Figure 8, both impedance plots of the LMO/GN-P and LMO/GN-F electrodes have the similar shape, which is composed of a semicircle in the high-to-medium frequency and a straight line in the low frequency. The slope angle of the straight line in the low-frequency region of the LMO/GN-F electrode is larger than that of the LMO/GN-P electrode, demonstrating that the LMO/GN-F electrode has a smaller Warburg impedance (*W*), resembling the solid-state diffusion of within the electrode [28,29]. The diameter of the semicircle in the high- to medium-frequency region for LMO/GN-F electrode is about 15 Ω, which is significantly smaller than that of the LMO/GN-P electrode (about 25 Ω), indicating that the LMO/GN-F electrode has a lower charge-transfer resistance (*R*_ct_) at the electrode/electrolyte interface. The equivalent circuits are inset in Figure 8. In addition to *W* and *R*_ct_, discussed above, *R*_Ω_ is the ohmic resistance representing the total resistance of the electrolyte, separator, and electrical contacts. *CPE* is the constant phase-angle element, involving double layer capacitance of the active materials. The enhancement of charge transfer and Li^+^ diffusion in combination with the lower aggregation of LiMn_2_O_4_ nanowires and the better volume change handling could lead to the superior electrochemical performance of the LMO/GN flexible film.

## 4. Conclusions

A flexible LMO/GN hybrid film was successfully prepared through a facile vacuum filtration and reduction process. Compared to the LMO/GN powder prepared by physical mixing, the designed LMO/GN film exhibits significantly enhanced electrochemical performance as cathodes in ReHABs. Benefited from the wrinkled surface and relatively large pore volume and specific surface area, the LMO/GN film could deliver reversible discharge capacities of 122.5, 119.3, 112.1, 101.1 and 86.6 mAh g^−1^ at 0.5, 1.0, 2.0, 4.0 and 8.0 C, respectively. Even after 200 charge-discharge cycles at 1.0 C, it could hold a high discharge capacity of 113.0 mAh g^−1^. The improvement of the electrochemical performance for LMO/GN film in ReHABs is mainly a result of the enhanced electronic conductivity and convenient Li^+^ transportation provided by graphene sheets, together with the positive effects of graphene in handling the aggregation and volume change of LMO nanowires.
